# Genetic Heterogeneity Correlated with Phenotypic Variability in 48 Patients with Cystic Fibrosis

**DOI:** 10.3390/jcm14155362

**Published:** 2025-07-29

**Authors:** Mădălina Andreea Donos, Lăcrămioara Ionela Butnariu, Dana Teodora Anton Păduraru, Alina Mariela Murgu, Cristina Rusu, Monica Cristina Pânzaru, Roxana Popescu, Elena Țarcă, Elena Cojocaru, Gabriela Ghiga, Laura Mihaela Trandafir

**Affiliations:** 1 Department of Mother and Child, Faculty of Medicine, “Grigore T. Popa” University of Medicine and Pharmacy, 700115 Iasi, Romania; madalina.donos@umfiasi.ro (M.A.D.); dana.anton@umfiasi.ro (D.T.A.P.); alina.murgu@umfiasi.ro (A.M.M.); gabriela.ghiga@umfiasi.ro (G.G.); laura.trandafir@umfiasi.ro (L.M.T.); 2 Department of Medical Genetics, Faculty of Medicine, “Grigore T. Popa” University of Medicine and Pharmacy, 700115 Iasi, Romania; abcrusu@gmail.com (C.R.); monica.panzaru@umfiasi.ro (M.C.P.); roxana.popescu@umfiasi.ro (R.P.); 3 Department of Surgery II—Pediatric Surgery, “Grigore T. Popa” University of Medicine and Pharmacy, 700115 Iaşi, Romania; elena.tuluc@umfiasi.ro; 4 Department of Morphofunctional Sciences I, Grigore T. Popa University of Medicine and Pharmacy, 700115 Iaşi, Romania; elena2.cojocaru@umfiasi.ro

**Keywords:** *CFTR*, variant, genotype, homozygous, heterozygous, NGS

## Abstract

**Background/Objectives**: Cystic fibrosis (CF) is a rare autosomal recessive genetic disease that has a progressive and multisystemic course. The spectrum and frequency of mutations in the gene encoding the cystic fibrosis transmembrane conductance regulator (CFTR) vary both in European countries and in other geographical regions. The aim of our retrospective study was to present the genetic variants identified in a group of 48 CF patients from the Moldova region (Romania), as well as to establish genotype–phenotype correlations. **Methods**: Genetic testing was initially performed for 38 *CFTR* mutations, and in heterozygous patients or those in whom no mutation was detected, *CFTR* gene sequencing (NGS) was performed. **Results**: The compound heterozygous genotype was identified in 26 (54.16%) of the patients (with one of the alleles being F508del), while 22 (45.83%) patients had the homozygous F508del genotype. The F508del variant was the most frequent (69.79%), followed by G542X (6.25%, 6/96). Several new variants were also identified that had not been reported in other studies from Romania (R1158X, K598*, R347H, c.2589_2599del, R496H, and CFTRdele2). Phenotypic manifestations in patients with *CFTR* class I, II, III and VII variants (homozygous and compound heterozygous) were more severe compared to those in patients with *CFTR* class IV, V and VI mutations, with the data obtained being consistent with those in the literature. Respiratory tract involvement was present in 77.08% of the patients, being more frequent in patients with the compound heterozygous genotype compared to the homozygous F508del genotype. Most patients had exocrine pancreatic insufficiency (EPI) (85.41%). Gastrointestinal manifestations included hepatocytolysis (66.66%) and biliary cirrhosis (0.41%). Meconium ileus was detected in 18.75% of patients, all with a compound heterozygous genotype. **Conclusions**: We compared the results obtained with data from the literature and correlated the detected *CFTR* variant (genotype) with the phenotypic manifestations, highlighting certain particularities present in some patients. Genetic testing allows for early diagnosis and adapted management, including personalized treatment for each patient. Identification of novel unclassified *CFTR* variants still remains a challenge for clinicians. NGS-based screening of heterozygous healthy carriers is important for both genetic counseling and prenatal diagnosis.

## 1. Introduction

Cystic fibrosis (CF) (ORPHA: 586; OMIM: 219700) is a rare autosomal recessive genetic disease caused by mutations in the cystic fibrosis transmembrane conductance regulator (*CFTR*) gene located on chromosome 7q31.2 (OMIM, 602421) [1].

CF is a progressive multisystem disease that impacts multiple organ systems, with the respiratory and digestive systems being the most commonly affected. It is characterized by recurrent sinusitis, bronchitis, and progressive lung disease, along with exocrine pancreatic insufficiency (EPI), diabetes, gastrointestinal manifestations, and male infertility due to congenital absence of the vas deferens (CAVD).

Pulmonary disease is the leading cause of morbidity and mortality in CF [2,3]. CF is the most common potentially lethal genetic disease in Caucasians, affecting roughly 1 in 3000–4000 live births. Although it is less common in African Americans (approximately 1 in 15,000–20,000) and even less common in Asian Americans, CF remains a significant health problem [1]. Approximately 1 in 25–30 Caucasians carry a pathogenic *CFTR* variant [4].

Mutations in the *CFTR* gene disrupt chloride and bicarbonate transport channel, leading to thick mucus, increased sodium absorption, and impaired mucociliary clearance. This predisposes individuals to inflammation, recurrent respiratory infections, and lung disease, which is the primary cause of morbidity and mortality in CF [5].

To date, 2121 *CFTR* variants have been reported in the Cystic Fibrosis Mutations Database, their distribution and frequency varying by region and ethnic group [6].

The most frequent CF mutation worldwide is c.1521_1523delCTT (F508del) (66.8% of CF cases). Its prevalence varies significantly by geographic region, with the highest frequencies observed in Northern European countries like Denmark (87.2%) and the lowest in Algeria (26.3%) [7,8,9]. Some populations have a lower frequency of F508del compared to other *CFTR* variants, and certain mutations are exclusively found in specific geographical areas [9].

Along with F508del, the most common variants are G542X (2.6%), N1303K (1.6%), G551D (1.5%), W1282X (1.0%), 1717-1G->A (0.83%), R553X (0.75%), 621+1G->T (0.54%), and R1162X (0.51%) [10,11,12].

In the Mediterranean area, the most prevalent CF variant is G542X, occurring at a frequency of 6.1%. The W1282X variant is also common in the Mediterranean region and North Africa, with the highest frequency observed in Ashkenazi Jewish populations in Israel (up to 40%) [12,13].

In contrast, the G551D variant is more frequently found in northwestern and central Europe [10,11]. The N1303K variant is most prevalent in Tunisia, at 17.2%, but it is also present in most western and Mediterranean countries [11,12,13,14].

Depending on their effect on the function, quantity, or stability of CFTR in the cell membrane, *CFTR* variants were initially classified into six classes [15,16].

Kris De Boeck and Margarida Amaral extended the initial classification to seven classes. Specifically, they divided the original class I mutations, which involve the absence of CFTR protein synthesis, into two groups: class I mutations, which are characterized by the lack of the CFTR protein and are susceptible to pharmacological rescue by read-through compounds, and class VII mutations, which are characterized by the lack of the *CFTR* mRNA transcript and which “cannot be rescued pharmacologically” [17,18].

Most class III mutations, which disrupt channel opening (or gating), are considered minimal function variants, but they can also be found in both classifications [19].

The phenotypic variability and severity of cystic fibrosis (CF) are influenced by several factors beyond *CFTR* gene mutations, including age, disease progression, environmental factors, and modifier genes [20,21]. Many studies have focused on correlating classes and *CFTR* variants with pancreatic, gastrointestinal, and reproductive manifestations [22,23].

In patients with symptoms suggestive of cystic fibrosis, genetic testing, specifically a DNA test for 38 *CFTR* gene mutations and/or *CFTR* sequencing, is crucial for confirming the diagnosis. Identifying the specific gene variant helps predict disease severity and guide therapeutic approaches.

The aim of our retrospective study was to present the genetic variants identified in a group of 48 patients with cystic fibrosis, as well as to establish genotype–phenotype correlations. The results obtained were compared with data from the literature. We also correlated the detected *CFTR* variant (genotype) with the phenotypic manifestations, highlighting certain particularities present in some patients.

## 2. Materials and Methods

We retrospectively analyzed a group of 48 patients diagnosed with cystic fibrosis, registered at the Children’s Emergency Clinical Hospital, St. Maria Iași, Romania, during the period 1990–2025. All patients came from the geographical region of Moldova. The clinical diagnosis of CF was carried out based on the clinical picture, which included symptoms of chronic sinopulmonary disease and/or gastrointestinal abnormalities/nutritional disorders associated with sweat test values higher than 80 mmol/L, performed by Nanoduct systems (Wescor, Logan, UT, USA). The modern Nanoduct sweat analysis system is an update to the classic method (Macroduct/Gibson–Cook method) of inducing sweating by iontophoresis with pilocarpine. The Nanoduct system represents a new, simple, and rapid method that allows for the collection of sweat and the measurement of sodium chloride levels via conductivity analysis. The normal values of this method are different from those of the classic Gibson–Cooke test; according to the Nanoduct user guide, the reference ranges for children under 16 years of age are as follows: normal—below 50 mmol/L NaCl; equivocal—50 to 80 mmol/L NaCl; and diagnostic of FC—above 80 mmol/L NaCl (1 mmol is equal to 1 mEq NaCl).

In patients with sweat chloride values greater than 80 mmol/L NaCl (performed twice to confirm results), as well as in patients who presented clinical manifestations suggestive of cystic fibrosis but in whom the sweat chloride values revealed equivocal values between 50 and 80 mmol/L, genetic testing was also performed (because we were unable to confirm the results obtained by Nanoduct with another method, e.g., Macroduct/Gibson–Cooke method).

Genetic testing was initially performed for 38 *CFTR* gene mutations, and in cases where a single mutation was detected (heterozygous status) or no mutation was detected, *CFTR* gene sequencing (NGS) was performed. The latter was performed in laboratories abroad, with the cost of the analysis being borne by the patients’ families since in Romania testing cannot be performed in state hospitals through the National Rare Diseases Program.

For the analysis of 38 *CFTR* mutations, DNA extraction was performed with the Invitrogen™ PureLink™ Genomic DNA Mini Kit (lot 1691968) from peripheral blood samples collected on EDTA according to the manufacturer’s instructions. Genotyping was performed using the IVD Cystic Fibrosis Genetic Assay kit (Nuclear Laser Medicine, Settala, Italy). The kit detects 38 mutations in the *CFTR* gene using a multiplex PCR amplification with biotinylated primers, followed by reverse hybridization on the strip and colorimetric detection (mutant allele/wild-type allele). Equipment used: Corbett Research thermal cycler (Corbett Life Science, Cambridge, UK).

*CFTR* gene sequencing was performed at laboratories abroad (Blueprint genetics and Invitae), using Next-Generation Sequencing (NGS) (Illumina Technology, Telangana, India).

The total genomic DNA was extracted from the biological sample using a bead-based method. Quantity of DNA was assessed using the fluorometric method. After assessment of DNA quantity, each qualified genomic DNA sample was randomly fragmented using non-contact, isothermal sonochemistry processing. The sequencing library was prepared by ligating sequencing adapters to both ends of DNA fragments. Ready sequencing libraries that passed quality control were sequenced using Illumina’s sequencing-by-synthesis method using paired-end sequencing (150 by 150 bases). Primary data analysis, converting images into base calls and associated quality scores, was carried out with the sequencing instrument using Illumina’s proprietary software (bcl2fastq), generating CBCL files as the final output. The pathogenicity of the identified gene variants was assessed according to the American College of Medical Genetics and Genomics and the Association for Molecular Pathology (ACMG/AMP) guidelines. For the interpretation of the variants identified in patients with CF, we also used the HGMD Professional and ClinVar databases.

The potential pathogenicity of novel missense variants was determined using three in silico prediction methods: PolyPhen-2 (http://genetics.bwh.harvard.edu/pph2/, accessed on 16 April 2025), PROVEAN (http://provean.jcvi.org/genome_submit_2.php?species?human, accessed on 16 April 2025), and MutationTaster (http://www.mutationtaster.org/ChrPos.html, accessed on 16 April 2025). In the case of the identification of CNVs, the Database of Genomic Variants and DECIPHER were analyzed, as well as the specialized literature, to evaluate their clinical relevance.

After the molecular classification of the mutations, for each patient and their family, we analyzed the data available in the observation files, including those obtained through family history and a genealogical tree. We analyzed the correlation between the patient’s phenotype and the detected gene variant (genotype) and highlighted the particularity present in some of the patients.

We also compared the obtained results with the data from the literature, highlighting both the similarities and the differences identified in the patients in the study group.

The study was conducted in accordance with the Declaration of Helsinki and approved by the Ethics Committee of the St. Mary’s Emergency Children Hospital, Iasi (certificate no. 31600/2023), and by the Research Ethics Committee of the University of Medicine and Pharmacy Grigore T. Popa Iasi (certificate no. 434 /2024).

For all children, informed consent was obtained from the parents, as well as from all adults who were clinically evaluated and who underwent genetic testing.

## 3. Results

This retrospective study included 48 (13.15%) patients with CF confirmed by molecular testing (DNA test for 38 *CFTR* mutations and/or *CFTR* gene sequencing) out of a total of 365 patients with suspected CF based on clinical manifestations correlated with iontophoresis values. The compound heterozygous genotype was identified in 26 (54.16%) of the patients (with one of the alleles being F508del), while 22 (45.83%) patients had the homozygous F508del genotype (Table 1).

**Figure 1:**
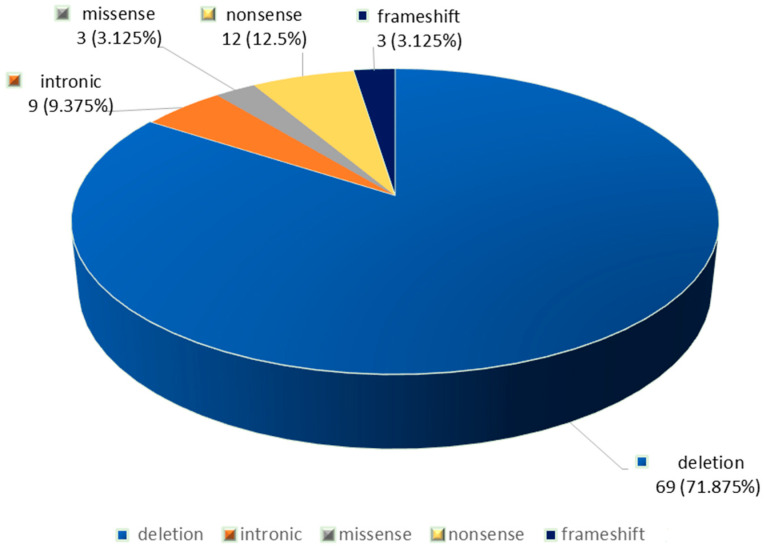
Types of *CFTR* variants detected in the group of patients with cystic fibrosis.

In the 48 patients, 96 *CFTR* gene variants were identified. The F508del variant was the most frequently detected (69.79%, 67/96), being present in 93.75% (45/48) of the patients, followed by nonsense (12.5%, 12/96) and intronic variants (9.375%, 9/96) (Figure 1). We also identified two non-F508del deletions (2.08%, 2/96), three t frameshift mutations (3.125%, 3/96), and three missense variants 3.125 (%, 3/96) (Table 1 and Figure 1).

Apart from the F508del variant, the most frequent variants detected were G542X (6.25%, 6/96), 621+1G>T (c.489+1G>T) (3.12%, 3/96), 1677delTA (3.12%, 3/96), 185+1G->T (c.53+1G>T) (3.12%, 3/96), 2184insA (2.08%, 2/96), c.917dupA p.(Asn306Lysfs*2) (2.08%, 2/96), and 3849G>A (c.3717G>A) (p.Arg1239=) (2.08%, 2/96) (Table 2).

The phenotypic manifestations detected in the 48 patients with CF are presented in Table 3.

Male patients (26/48, 54.16%) were more numerous than female patients (22/48, 45.83%) (Table 2). Respiratory tract involvement was present in 77.08% (37/48) of the patients, being more frequent in patients with a compound heterozygous genotype (22/26, 84.61%) compared to those with the homozygous F508del genotype (15/22, 68.18%). Recurrent upper respiratory tract infections (R-URTIs) (23/48, 47.91%) were more common than bronchiectasis (14/48, 29.16%). The most common colonization of patients was with *Staphylococcus aureus* (23/48, 47.91%) and *Pseudomonas aeruginosa* (18/48, 37.5%) (Table 3).

Exocrine pancreatic insufficiency (EPI) was detected in 41/48 patients (85.41%) with CF (22 homozygotes and 19 compound heterozygotes) (Table 3). Other CF-related gastrointestinal (GI) manifestations included hepatocytolysis (32/48, 66.66%) and biliary cirrhosis (5/48, 10.41%) (Table 3). In 93.75% (45/48) of cases, there was growth failure, and the most frequent metabolic disorders were 25-OH vitamin D deficiency (27/48, 56.25%), hepatic steatosis (11/48, 22.91%), and cystic fibrosis-related diabetes (CFRD) (6/48, 12.5%) (Table 3). Meconium ileus was present in 18.75% (9/48) of cases, subocclusive syndrome was detected in 6.25% (3/48) of cases, and 12.5% (6/48) of patients presented with rectal prolapse (Table 3). The most frequent nonspecific, atypical manifestations detected in patients with CF were neuropsychiatric disorders (11/48, 22.91%) and cardiac involvement (10/48, 20.83%). Other manifestations were congenital adrenal hyperplasia (CAH) (2/48, 4.16%), congenital anomalies of the kidney and urinary tract (CAKUT) (2/48, 4.16%), gluten-related disorders (GRDs) (2/48, 4.16%), and growth hormone deficiency (GHD) (1/48, 2.08%).

## 4. Discussions

Molecular testing in the 48 patients identified 96 *CFTR* variants, the most frequent being F508del (67/96, 69.79%). F508del (class I mutation) was identified in 93.75% (45/48) of the patients: 22 (45.83%) cases with a homozygous genotype and 26 (54.16%) patients with a compound heterozygous genotype (Table 1 and Figure 1). In three patients (6.25%) with a compound heterozygous genotype, two different allelic variants, other than F508del, were identified (Table 1).

To date, there are few studies that attest to the prevalence of *CFTR* mutations in the Romanian population. The frequency of the F508del variant varies in different studies.

Frentescu et al. [24] analyzed 128 Romanian patients with CF, of whom 56.3% carried at least one F508del allele (35.93% with a homozygous genotype) [24].

In another study, Apostol et al. [29] identified the F508del variant in 89.5% of a cohort of 19 patients with CF, all of whom came from the southern region of Romania [29].

In 203 of the 355 Romanian children with CF included in the Romanian Cystic Fibrosis database, Dobre et al. [30] identified a frequency of 62% for the F508del variant [30]. The authors showed that most of the patients were F508del homozygotes (44.3%), while 35.9% of the patients were compound heterozygotes with at least one F508del mutation [30]. The majority of patients came from the Timis, Iasi (Moldova), and Bucharest counties [30]. In a 1997 study, Popa et al. [31] reported the frequency of the F508del variant in the Romanian population as 25% [31].

It is possible that the frequency of the F508del variant in the Romanian population is underestimated in some studies, and the differences that exist between the data reported by different authors could have several causes. These could include the small number of patients analyzed in some studies and the variation in *CFTR* mutations in different geographical regions of the country, as well as the lack of uniform criteria for patient selection or clinical diagnostic difficulties. Future studies that will include a much larger number of patients with cystic fibrosis from different regions of the country will most likely provide new data related to the prevalence of the F508del variant in Romania.

Also, access to genetic testing plays a key role in confirming the diagnosis of CF. In Romania, free testing through the National Rare Diseases Program only includes screening for 38 mutations of the *CFTR* gene, while the cost of *CFTR* gene sequencing is borne by the patient’s family. Thus, many of the patients heterozygous for 1 of the 38 tested mutations, who have clinical manifestations characteristic of CF and positive iontophoresis, remain undiagnosed, because the parents cannot afford the cost of *CFTR* gene sequencing.

The prevalence of the F508del variant in Europe is between 65 and 70%, with large variations between southern Europe (30–35%) compared to northern Europe (87%) [32]. We consider that the frequency of the F508del variant in the patients in our study (69.79%) is consistent with European data.

In Table 2, we compare the frequency of *CFTR* variants identified in our study with those presented in other European studies.

In a 2024 study that analyzed *CFTR* variant diversity across ancestries characterized using 454,727 UK biobank whole exome sequences, Ideozu et al. [33] identified 3192 (76.1%) *CFTR* variants in the European population. The F508del variant represented 90% of the CF-causing variant, compared to other ancestries, where the frequency of this variant was lower [33]. We believe that the results of our study (in which we identified 96 *CFTR*-causing variants, of which the F508del variant represented 69.79% (67/96), being present in 93.75% (45/48) of CF patients) are consistent with those reported by Ideozu et al.

In a study that included 61 patients with CF from the Republic of Moldova, Sciuca et al. [25] identified the frequency of the F508del variant as 54.71% (70/122), similar to those reported for the Romanian population in the studies of Frentescu et al. (56.3%) [24] and Dobre et al. (62%) [30].

In another study that included 192 CF patients from the Murcia region (Spain) (southwestern Europe), Rueda-Nieto et al. [7] identified the F508del variant in 58.3% of patients (27% homozygotes and 73% compound heterozygotes), representing 37% of all allelic variants identified [7].

The frequency of the F508del variant was 83.19% in the study by Kasmi et al. [26] in 116 patients from Albania [26]. The results of our study and the Albanian study (both countries from southeastern Europe) showed (a fact already known) that there are variations in the prevalence of the F508del variant in southeastern Europe [26,27].

### Genotype–Phenotype Correlations in Patients with Cystic Fibrosis

Genotype–phenotype correlations in CF, the intervention of modifier genes, and the interaction with environmental and epigenetic factors facilitate our understanding of the wide spectrum of disease manifestations, which can vary from single to multisystemic involvement and from mild to severe disease [15,20,34].

Phenotypic manifestations in patients with *CFTR* class I, II, III and VII variants (homozygous and compound heterozygous) were more severe compared to those of patients with *CFTR* class IV, Vand VI variants, the data obtained being consistent with those in the literature.

**Figure 2:**
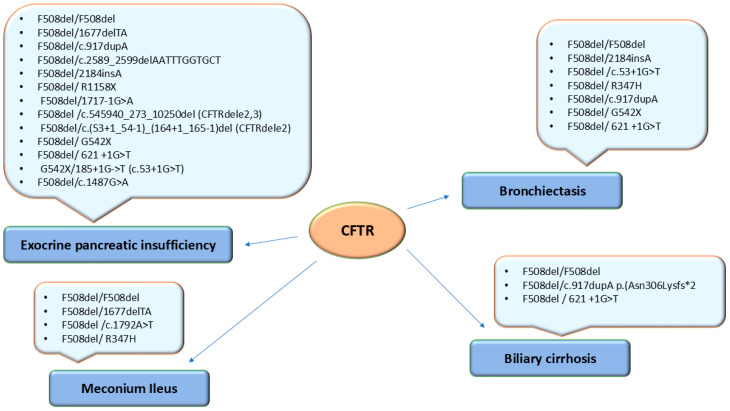
Genotype–phenotype correlation in 48 patients with cystic fibrosis.

Meconium ileus was present in 9/48 patients (18.75%), of which 6 (12.5%) had homozygous F508del/F508del mutations and 3 (6.25%) had compound heterozygous mutations: F508del/1677delTA, F508del/c.1792A>T, and F508del/R347H (Figure 2).

The prevalence of meconium ileus in CF patients in our study (18,75%) is consistent with data from the literature (the prevalence of meconium ileus varies in different registries between 13 and 21%). In their study, Dupui et al. [23] found that the presence of meconium ileus is linked to more severe CF disease with exocrine pancreatic insufficiency (EPI) in individuals carrying the G542X and F508del variants [23].

Bronchiectasis was present in 5/22 (22.72%) homozygous F508del/F508del patients and in 9/26 (34.61%) compound heterozygous patients: F508del/2184insA—2 cases; F508del/c.53+1G>T—1 case; F508del/R347H—1 case; F508del/c.917dupA—1 case; F508del/G542X—2 cases; and F508del/621 +1G>T—2 cases (Figure 2).

The majority of patients (41/48) with CF had exocrine pancreatic insufficiency (EPI) (85.41%). This finding aligns with data from the literature according to which over 85% of CF patients develop EPI (European Cystic Fibrosis Society Patient Registry) [32].

The evaluation of pancreatic function was performed by determining fecal pancreatic elastase-1 (FE-1) in two stool samples, considering pancreatic insufficiency at FE-1 values < 200 µg/g (European Cystic Fibrosis Society (ECFS) standards for the care of people with CF) [32]. In the case of our patients, most had severe pancreatic insufficiency, with FE-1 values below 100 µg/g [32].

EPI was identified in 22 F508del/F508del homozygotes and 19 compound heterozygotes: F508del/1677delTA—3 cases; F508del/c.917dupA—2 cases; F508del/c.2589_2599delAATTTGGTGCT—1 case; F508del/2184insA—2 cases; F508del/R1158X—1 case; F508del/1717-1G>A—1 case; F508del/c.545940_273_10250del (CFTRdele2;3)—1 case; F508del/G542X—3 cases; F508del/c.621+1G>T—3 cases; G542X/185+1G->T (c.53+1G>T)—1 case; F508del/c.(53+1_54-1)_(164+1_165-1)del (CFTRdele2)—1 case; and F508del/c.1487G>A, p.(Trp496Ter) (W496*)—1 case (Figure 2).

Biliary cirrhosis was identified in three (6.25%) patients: two patients with compound heterozygous genotypes—F508del/c.917dupA p.(Asn306Lysfs*2) and F508del/621+1G>T—and three patients with a homozygous F508del/F508del genotype (Figure 2).

Five (10.41%) patients died due to complications (age of death ranging between 2 and 8 months of life): three (6.25%) with the homozygous genotype F508del/F508del and two (4.16%) patients with the compound heterozygous genotype F508del/1677del TA. The main cause of death was sepsis with multiple organ dysfunction, and one patient was diagnosed with intestinal volvulus with ileal loop necrosis, meconium peritonitis, common mesenteric thrombosis, and severe sepsis with cloacal *Enterobacter* and *P. aeruginosa*.

The G542X variant (class I, nonsense) was detected in six (6.25%) compound heterozygous patients, being the most frequent mutation detected in our study, after the F508del variant (Table 1): F508del/G542X—three cases and G542X/185+1G->T (c.53+1G>T)—three cases.

G542X is common in Mediterranean countries (6.1–8%) [11,12]. The phenotype of patients with the F508del/G542X compound heterozygous genotype is severe, involving meconium ileus, EPI, hepatobiliary involvement, and congenital absence of the vas deferens (CAVD) [35,36,37,38]. In the case of the three patients with CF and the F508del/G542X genotype in our study, the phenotype was severe and included growth and development delay, recurrent upper respiratory tract infections (R-URTIs), recurrent pneumonia, bronchiectasis, chronic coinfection with *P.aeruginosa* and *Staphylococcus aureus*, hepatocytolysis, hepatic steatosis, and vitamin D deficiency. Two of the patients presented with rectal prolapse and EPI. CFRD was detected in one of the patients.

In other three patients with CF, the compound heterozygous genotype G542X/185+1G->T (c.53+1G>T) was detected (Table 1). All three patients had a similar severe phenotype causing recurrent pneumonia, chronic coinfections with *P.aeruginosa* and *Staphylococcus aureus,* anemia, and cystic fibrosis-associated liver disease (CFLD). Only one of the patients developed EPI and rectal prolapse associated with immune deficiency and atopic dermatitis. The *CFTR* c.53+1G>T variant (class V, splice donor) is located at a canonical splice site and is predicted to affect mRNA splicing, resulting in an abnormal protein (either by exon skipping or by inclusion of intronic material).

The *CFTR* c.53+1G>T variant is classified as pathogenic and is present in the ClinVar database (Variation ID: 53988) [39], having been reported in patients with CF in various studies in the literature (PMID: 22658665, 16596947, 23276700, 31126253) [28,40,41,42].

Three other patients with CF in the study group were compound heterozygous F508del/621+1G>T (c.489+1 G>T) (Table 1). In all three patients, the phenotype was severe, with clinical manifestations including recurrent pneumonia, EPI, growth delay, and vitamin D deficiency. Other manifestations were inflammatory bowel disease (IBD) (one case), hepatocytolysis, pituitary dwarfism (one case), and intellectual disability (one case).

The *CFTR* variant 621+1G>T (c.489+1 G>T) (class I, splice donor) is present in the ClinVar database (Variation ID: 38799) [39] and is classified as pathogenic. It has been reported in the literature in patients whose phenotype included bronchiectasis, hereditary pancreatitis, and CFTR-related disorders (CFTR–RD) [https://cftr2.org/mutations_history, accessed on 29 March 2025] [43,44,45,46,47].

The 1677delTA variant (class II, nonsense) was detected in three compound heterozygous patients, with the other allele being F508del (Table 1). The 1677delTA variant (a 2bp deletion in exon 10 of the *CFTR* gene) is common in the Black Sea basin (the highest frequency being detected in Georgia) and is associated with a severe CF phenotype, with a high rate of early mortality in homozygotes, and possibly with an increased risk of meconium ileus [48,49].

Our results are consistent with those in the literature, with the phenotype of the three patients with the compound heterozygous genotype F508del/1677delTA being severe, two of them dying in the first months of life, and the other patient developing meconium ileus, with the subsequent evolution being severe. The F508del/1677delTA variant was previously reported in a patient from Romania by Frentescu et al. [24] and by Sciuca et al. [25] in a patient from the Republic of Moldova.

The compound heterozygous genotype F508del/2184insA was identified in two of the patients with CF (Table 1) and was associated with a severe disease phenotype. Thus, one of the patients underwent associated growth failure, recurrent respiratory infections, bronchiectasis, chronic colonization with *P.aeruginosa*, and EPI, and the other two patients also presented chronic coinfection with *Moraxella catarrhalis* and *Burkholderia cepacia*, renal lithiasis, dyslipidemia, immune deficiency, and CFRD.

The 2184insA c.2052dup (p.Gln685fs) (Q685fs) variant (class I, frameshift) was previously reported by Frentescu et al. [24] in a patient with CF from another geographical region of Romania (Cluj) other than Moldova and in four patients from the Republic of Moldova in a study by Sciuca et al. [25].

The pathogenic *CFTR* c.2052dupA (p.Gln685ThrfsTer4) (Q685Tfs*4, or 2184insA) variant (most likely originating from the former Galicia community) is present in the ClinVar database (Variation ID: 35838) [39] and has been reported in the literature in several individuals affected by FC with exocrine pancreatic insufficiency (EPI) [40,50,51,52,53,54,55,56,57].

In the other two patients with CF, the compound heterozygous genotype F508del/c.917dupA was detected (Table 1). In both cases, the phenotype was severe and included EPI associated with CFLD (hepatocytolysis and liver cirrhosis), a history of recurrent pneumonia, bronchiectasis, immune deficiency, and vitamin D deficiency. Both patients had iontophoresis values above 100 mmol/L NaCl. The *CFTR* variant (NM_000492.4) c.917dupA (p.Asn306LysfsTer2) (p.N306Kfs*2) is a missense mutation (class I) that is not found in the ClinVar and GnomAD databases or others, and it has not been reported in previous studies in patients from Romania, nor in other studies in the literature.

The compound heterozygous genotype F508del/3849G->A (c.3717G>A) (p.Arg1239=) was detected in two patients in the study group, in whom clinical manifestations included growth failure, R-URTIs, recurrent pneumonia, chronic colonization with *P.aeruginosa* and *Staphylococcus aureus,* hepatocytolysis, dyslipidemia, and immune deficiency without pancreatic involvement.

The *CFTR* variant NM_000492.4: c.3717G>A (3849G->A) (p.Arg1239=) (class V, splice donor) is present in the ClinVar (Variation ID: 53791) [39] and GnomAD databases (allele frequency in the general population: 0.00001), being classified as pathogenic [52]. This variant has been reported in several other studies in multiple individuals with CF and pancreatic insufficiency [58,59,60,61,62]. It was also reported in an individual with unilateral CAVD by Akinsal et al. [63] and as a variant with a lower risk of developing CFRD by Adler et al. [64].

The *CFTR* variant c.2589_2599del (p.Ile864fs) (I864fs) (class I, frameshift) was detected in a patient with a compound heterozygous genotype (the other allele being F508del). He presented with severe clinical manifestations, which included growth failure, recurrent pneumonia, *Staphylococcus aureus* chronic infection, rectal prolapse, and EPI. This mutation is classified as pathogenic and is present in the ClinVar database (Variation ID: 53516) [39] and absent in the GnomAD database, and it is reported in several studies in the literature (PMID: 30979683, 30888834, 30548586) [65,66,67].

Two other novel variants (not previously reported in the Romanian population) associated with meconium ileus were K598*(c.1792A>T) (p.Lys598Ter) and R347H. Both patients had a compound heterozygous genotype, with the other allele being F508del (Table 1).

The patient with the K598*(c.1792A>T) (p.Lys598Ter) variant (class V, nonsense) had consanguineous parents, who had a history of another child with CF, who died at the age of 5 weeks. The clinical evolution involved recurrent respiratory infections, growth failure, maldigestion with secondary anemia, hypoproteinemia, and severe deficiency rickets. This variant is present in the ClinVar (Variation ID: 1705874) [39] and GnomAD databases and is reported as pathogenic in a single study by Claustres et al. [68].

The patient in whom the c.1040G>A (p.Arg347His) (R347H) variant (class III, missense) was identified presented with, in addition to meconium ileus, recurrent respiratory infections, colonization with *Staphylococcus aureus*, bronchiectasis, and vitamin D deficiency. This missense variant is present in the ClinVar database (Variation ID: 7182) [39] and is classified as pathogenic. It has been reported in several studies in patients with CF who have developed pancreatitis (PMID: 30726326, 22658665, 27086061) [36,42,69].

In two other patients who developed EPI, the variants R1158X (c.3472C>T) (class I, nonsense) and 1717-1G>A (class VII, splice acceptor) were identified, both being novel variants that had not previously been reported in the population of Romania or the Republic of Moldova (Table 2).

Both patients had a compound heterozygous genotype, with the other allele being F508del (Table 1). Clinical manifestations in the case of the R1158X variant included, in addition to EPI, growth failure, R-URTIs, recurrent pneumonia, hepatocytolysis, immune deficiency, and adrenogenital syndrome (CAH).

The nonsense variant R1158X c.3472C>T (p.Arg1158Ter) is present in the ClinVar (Variation ID: 7144) [39] and GnomAD databases, is classified as pathogenic, and has been reported in several studies in patients with CF (PMID: 22658665, 23974870, 31672438) [40,47,70].

In addition to EPI, the clinical manifestations in the patient with the 1717-1G>A (c.1585-1G>A) variant (IVS10, G-A, -1) included growth failure, chronic diarrhea, recurrent pneumonia, and vitamin D deficiency. The intronic variant 1717-1G>A (c.1585-1G>A) is present in the ClinVar (Variation ID: 7112) [39] and GnomAD databases and is classified as pathogenic. It is considered one of the most common variants associated with CF and has been reported in several studies in the literature [40,71,72,73].

In two patients with CF who presented with neonatal cholestasis syndrome, the *CFTR* variants c.(53+1_54-1)_(164+1_165-1)del (CFTRdele2) and c.54-5940_273+10250del (CFTRdele2,3 (21kb)) were identified, in both cases with a compound heterozygous genotype, with the other allele being F508del (Table 1).

In the case of the first patient, clinical manifestations included growth failure, EPI, IBD (chronic diarrhea), CFLD (hepatocytolysis, acute liver failure), hyper IgE syndrome, and immune deficiency. The c.(53+1_54-1)_(164+1_165-1)del variant (class I) has not been reported in previous studies in the Romanian population and is present in the ClinVar database (Variation ID: 1705984) [39], being classified as pathogenic [74].

For the second patient in whom the variant c.54-5940_273+10250del (CFTRdele2,3 (21kb)) (class VII) was identified, the clinical manifestations included, in addition to delayed meconium elimination, hemolytic anemia and hypoproteinemia. This variant was previously reported in Romania by Frentescu et al. [24], as well as in the Republic of Moldova by Sciuca et al. [25] (Table 2). This variant (CFTRdele2,3) involves the deletion of exons 2 and 3 of the *CFTR* gene and can be found in the ClinVar database (Variation ID: 66105) [39], and it is classified as pathogenic, having been reported in several studies in the literature (PMID: 24624459, 23974870) [75,76].

In a patient with CF whose phenotype included short stature, chronic diarrhea, chronic rhinosinusitis, chronic infection with *P.aeruginosa* and *Staphylococcus aureus,* EPI, hepatocytolysis, CFRD, dyslipidemia, immune deficiency, and vitamin D deficiency, the compound heterozygous genotype F508del/c.1487G>A, p.(Trp496Ter) (W496*) was detected. The *CFTR* variant c.1487G>A, p.(Trp496Ter) (W496*) (class V, nonsense) is present in the ClinVar database (Variation ID: 53265) [34], is classified as pathogenic, and is reported in the literature in several studies of patients with CF [51,52,53,54,55,56,57,58,59,60,61,62,63,64,65,66,67,68,69,70,71,72,73,74,75,76,77].

## 5. Conclusions

Although it included a relatively small number of patients, our retrospective study revealed that genetic heterogeneity correlates with phenotypic variability in cystic fibrosis.

The F508del variant was the most frequently (69.79%) detected, a similar frequency being reported in the literature for other European countries (65–70%). We identified both new variants and variants already reported in other studies, both for the Romanian population and for other European countries or other geographical regions.

Genetic testing has allowed for early diagnosis and adapted management (including personalized treatment) for each patient.

The particular phenotypic manifestations of certain patients could be explained by the intervention of phenotype-modifying factors (genes or environmental factors), including epigenetic regulation of *CFTR* gene expression.

The identification of a large number of unclassified *CFTR* variants by molecular testing (NGS) can be challenging and, at the same time, of interest and relevance to clinicians. NGS-based screening for the identification of healthy heterozygous carriers in affected families is important for genetic counseling and calculating the risk of recurrence in offspring, as well as for prenatal diagnosis.

## Figures and Tables

**Table 1:** *CFTR* variant detected in the group of 48 patients with cystic fibrosis.

Allele1	Allele 2	No of Cases
F508del	F508del	22
F508del	621+1G>T (c.489+1 G>T)	3
F508del	G542X	3
F508del	1677delTA	3
G542X	185+1G->T (c.53+1G>T)	3
F508del	2184insA (c.2052dup) (p.Gln685fs) (Q685fs)	2
F508del	c.917dupA p.(Asn306Lysfs*2)	2
F508del	c.3849G->A (c.3717G>A) (p.Arg1239=)	2
F508del	R1158X	1
F508del	K598*(c.1792A>T) (p.Lys598Ter)	1
F508del	c.1040G>A (p.Arg347His) (R347H)	1
F508del	c.54-5940_273+10250del (CFTRdele2,3 (21kb))	1
F508del	1717-1G>A	1
F508del	c2589_2599delAATTTGGTGCT c.2589_2599del (p.Ile864fs)(I864fs)	1
F508del	c.1487G>A, p.(Trp496Ter)	1
F508del	c.(53+1_54-1)_(164+1_165-1)del (CFTRdele2)	1

**Table 2:** The spectrum of *CFTR* variants identified in CF patients in our study and their frequency, compared to the results of other European studies.

*CFTR* Variant	No. Allele (%) (Our Study)	Frentescu et al. [24] (Romania)	Sciuca et al. [25] (Rep. Moldova)	Kasmi et al. [26] (Albania)	Petrova et al. [27] (Bulgaria	Křenková et al. [28] Czech Rep.)	Ruenda -Nieto et al. [7] (Spain)
F508del	67 (69.79%)	144 (56.3%)	79 (57.4%)	203 (83.19%)	154 (55%)	809 (67.42%)	142 (37.0%)
G542X	6 (6.25%)	10 (3.9%)	4 (3.3%)	4 (1.63%)	11 (3.93%)	24 (2%)	31 (8.1%)
621+1G>T (c.489+1G>T)	3 (3.12%)	2 (0.8%)	1 (0.8%)	6 (2.45%)	4 (1.43%)	5 (0.43%)	1 (0.3%)
1677delTA	3 (3.12%)	1 (0.4%)	1 (0.8%)	-	3 (1.07%)	-	-
185+1G ->T (c.53+1G>T)	3 (3.12%)	-	1 (0.8%)	-	-	2 (0.17%)	-
2184insA	2 (2.08%)	-	4 (3.3%)	-	8 (2.89%)	5 (0.42%)	-
c.917dupA p.(Asn306Lysfs*2)	2 (2.08%)	-	-	-	-	-	
3849G>A (c.3717G>A) (p.Arg1239=)	2 (2.08%)	-	2 (1.6%)	**-**	-	-	1 (0.3%)
R1158X (c.3472C>T)	1 (1.04%)	-	-	1 (0.40%)	1 (0.36%)	1 (0.08)	1 (0.3%)
K598* (c.1792A>T) (p.Lys598Ter)	1 (1.04%)	-	-	-	-	-	-
R347H (c.1040G>A)	1 (1.04%)	-	-	-	-	1 (0.08%)	-
c.54-5940_273+10250del (CFTRdele2,3 (21kb)	1 (1.04%)	4 (1.6%)	2 (1.6%)	1 (0.40%)	2 (0.71)	69 (5.75%)	-
1717-1G>A (c.1585-2A>T)	1 (1.04%)	1 (0.4%)	-	-	-	4 (0.33)	1 (0.3%)
c2589_2599delAATTTGGTGCT c.2589_2599del (p.Ile864fs)	1 (1.04%)	-	-	-	-	1 (0.08)	-
R496H (c.1487G>A) p.(Trp496Ter)	1 (1.04%)	-		-	-	-	-
c.(53+1_54-1)_(164+1_165-1)del (CFTRdele2)	1 (1.04%)	-	-	-	-	-	-
No total allele	96	256	122	244	277	1200	384

**Table 3:** The phenotypic manifestations in the 48 patients with CF correlated with the homozygous and compound heterozygous genotypes.

Criteria	Homozygous No. (%)	Compound Heterozygous No. (%)
Female	12/22 (54.5%)	12/26 (46.15%)
Male	10/22 (45.5%)	14/26 (53.84%)
*Respiratory manifestations*		
Bronchiectasis	5/22 (22.72%)	9/26 (34.61%)
R-URTIs ^a^	10/22 (45.45%)	13/26 (50%)
*Gastrointestinal and nutritional* *manifestations*		
Hepatocytolysis	14/22 (63.63%)	18/26 (69.23%)
Biliary cirrhosis	3/22 (13.63%)	2/26 (7.69%)
Liver fibrosis	1/22 (4.54%)	-
Gallbladder stones	2/22 (9.09%)	1/26 (3.84%)
EPI ^b^	22/22 (100%)	19/26 (73.07%)
CF-related pancreatitis	1/22 (4.54%)	-
CF-related GI manifestations	-	1/26 (3.84%)
Growth failure	19/22 (86.36%)	26/26 (100%)
*Metabolic manifestations*		
Dyslipidemia	2/22 (9.09%)	3/26 (11.53%)
Hepatic steatosis	5/22 (22.72%)	6/26 (23.07%)
CFRD	3/22 (13.63%)	3/26 (11.53%)
25-OH vitamin D deficiency	13/22 (59.09%)	14/26 (53.84%)
*Surgical manifestations*		
Meconium ileus	6/22 (27.27%)	3/26 (11.53%)
Subocclusive syndrome	2/22 (9.09%)	1/26 (3.84%)
Rectal prolapse	2/22 (9.09%)	4/26 (15.38%)
*Renal and urological manifestations*		
Urolithiasis	-	1/26(3.84%)

^a^ It includes chronic colonization with *Staphylococcus aureus*, *Pseudomonas aeruginosa* and *Burkholderia cepacia*, *Moraxella catarrhalis,* and *Acinetobacter*; R-URTIs: recurrent upper respiratory tract infections; CF-related GI manifestations: cystic fibrosis gastrointestinal manifestations; CFRD: cystic fibrosis-related diabetes;; EPI ^b^: exocrine pancreatic insufficiency defined by FE-1 levels < 200 μg/g (twice); 95% of patients diagnosed with EPI had FE-1 levels < 100 μg/g (widely considered as severe EPI).

## Data Availability

Data are contained within the article.

## Abbreviations

CF: Cystic fibrosis; CFTR: cystic fibrosis transmembrane conductance regulator; R-URTIs: Recurrent upper respiratory tract infections; CFLD: Cystic Fibrosis-associated liver disease; CFRD: Cystic Fibrosis-Related Diabetes; IBD: Inflammatory bowel disease; CFTR–RD: CFTR-related disorders; EPI; Exocrine pancreatic insufficiency.
